# Fixed lingual orthodontic retainer with bilateral missing lateral incisors produced in PEEK material using CAD/CAM technology

**DOI:** 10.4317/jced.58035

**Published:** 2021-06-01

**Authors:** Khaled Aboulazm, Constantin von See, Ahmed Othman

**Affiliations:** 1 Assoc. Prof., MSc, PhD. Chairman of orthodontic department in Pharos Private University-Alexandria- Egypt; 2 Univ. Prof., MSc. Director of digital technologies in dentistry and CAD/CAM department-Danube Private University-Krems-Austria; 3 Ass. Prof. Dr, MSc. Orthodontic researcher in digital technologies in dentistry and CAD/CAM department-Danube Private University-Krems-Austria

## Abstract

**Background:**

The aim of this investigation is to evaluate the feasibility of digital workflow for lingual fixed retainer based on digital intraoral scan and appliance production from Polyetheretherketone (PEEK) material for clinical consideration.

**Material and Methods:**

Fully virtual lingual retainer with bilateral missing lateral incisors was designed using inlab software (Dentsply Sirona, Pennsylvania, USA). The designed retainer was produced in PEEK material and clinically adhered to lingual surfaces of the lower front teeth.

**Results:**

Lingual retainer was successfully fabricated by full digital workflow and produced from PEEK material for clinical usage.

**Conclusions:**

Although full digital workflow can be clinically used for production of prefabricated lingual retainer, however further software adaptions are required for improvement of the orthodontic workflow.

** Key words:**PEEK, CAD/CAM, digital orthodontics, lingual retainer.

## Introduction

The usage of CAD/CAM technology in dentistry has been enrolled for more than 30 years ago (1). Studies were conducted to evaluate the involvement of digital workflow in clinical orthodontics and research fields (2,3). The reduction of chair time offered by single visit is considered one of the main advantages in digital dentistry (3). However, the expenses of software and hardware still limit CAD/CAM clinical usage nowadays. Indirect orthodontic bonding, lateral cephalometric tracing, model analysis and appliances designing are nowadays being digitally implemented (4-7). Orthodontic fixed functional appliances can be digitally designed and produced using the digital workflow for clinical usage and consideration (6).

Polyetheretherketone (PEEK) material is a by polycyclic, aromatic, thermoplastic polymer that is semi-crystalline and has a linear structure. Also, for dental manufacturing and incorporation it has acceptable mechanical and electrical properties such as resistance to high temperature and hydrolysis (8). The usage of digitally designed and produced fixed lingual retainer in PEEK material has not been clinically investigated, accordingly the feasibility of producing a retainer via CAD/CAM will be investigated.

Material and methods

**Figure 1:**
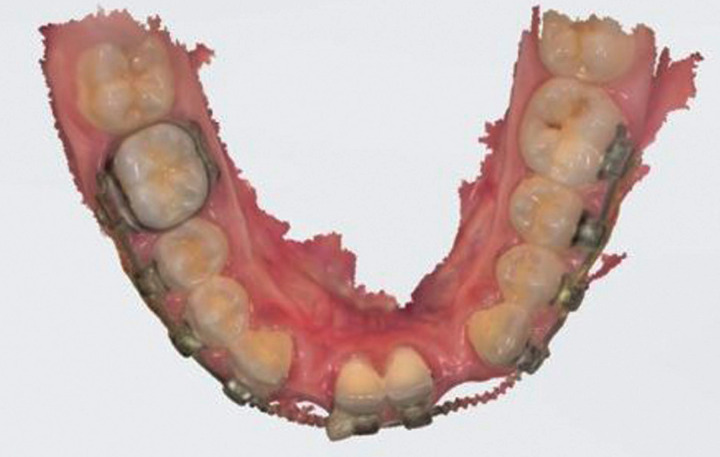
Digital impression of bracket bonded lower dental arch using Trios 3 intraoral camera (3Shape, Copenhagen, Denmark).

**Figure 2:**
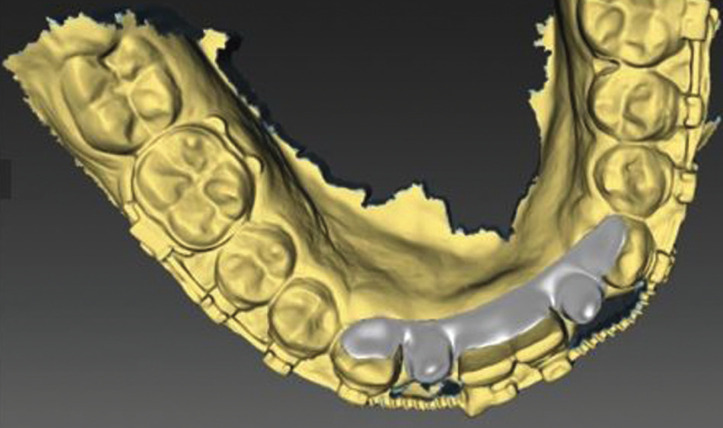
Digitally designed lingual retainer with missing laterals incisors using Inlab software (Sirona, Pennsylvania, USA).

**Figure 3:**
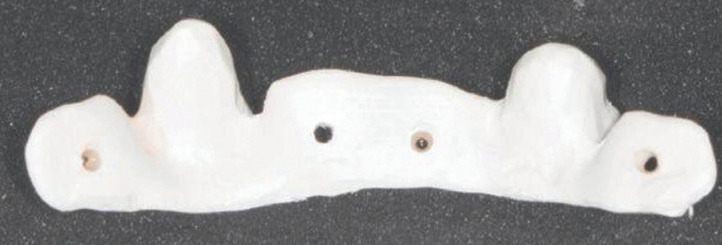
Lingual retainer with attached lateral incisors and retentive holes produced via CAD/CAM technology using PEEK material.

**Figure 4:**
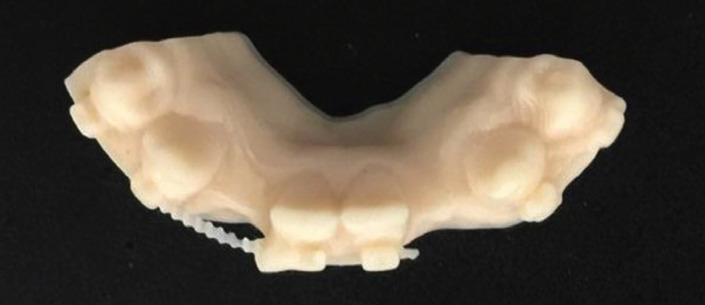
3D printed model for pre-evaluation of the produced retainer.

A digital impression using Trios 3 (3Shape, Copenhagen, Denmark) for an orthodontic patient with missing lower bilateral lateral incisors was recorded after orthodontic finishing (Fig. 1). The digital impression was imported as an .stl file into Inlab software (Sirona, Pennsylvania, USA) to design the lingual retainer (Fig. 2). The resulted design was exported as an .stl file and sent to the dental lab for milling production using a MCX5 (Dentsply Sirona, Pennsylvania, USA) from PEEK material. The restored lateral incisors were not welded nor soldered to the lingual retainer but it was produced as one-unit appliance to eliminate possible weak points and enhance the design mechanical properties (Fig. 3). For controlling purposes, 3D model was printed (Formlabs, Massachusetts, USA) preoperatively for evaluation the digitally fabricated retainer precision (Fig. 4).

**Figure 5:**
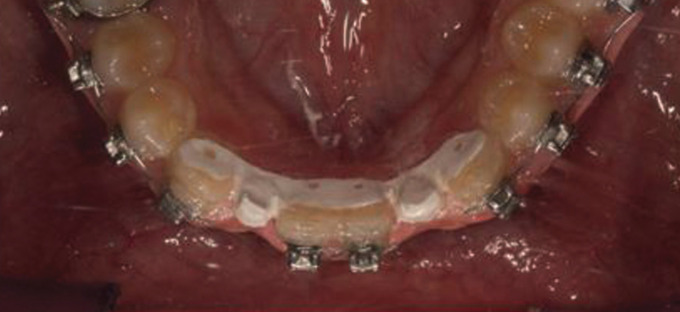
Produced modified lingual retainer adhered to the lingual surfaces of lower central incisors and canines.

After production of the retainer, etch and bonding technique was performed using a dual cured resin modified glass ionomer cement (GC, Tokyo, Japan). The resin-modified glass-ionomer cement showed the lowest enamel discolouration and higher mechanical characteristics (9,10). Accordingly, in this investigation the G-CEM capsule was used as an adhesive resin for the designed retainer. Before adherence, the lingual surfaces of lower central incisors and canines were cleaned, polished and sandblasted with 50µm (Skysea, China) to eliminate any adhered bacteria and biofilms. The cement base and catalyst were mixed following the manufacturer’s instructions to endure homogeneity. The 3M Espe Elipar device (3M, Minnesota, USA) was used for light curing with wave length LED technology in a spectrum range of 430nm – 480nm. Light device LED was applied on the fixed retainer for the cement curing following manufacturer’s instructions for 2-4 seconds alternatively to allow self-cure followed by removing material excess and curing each tooth for 10 more seconds (Fig. 5).

## Results

The .stl file for the designed retainer was forwarded digitally to the dental Lab to produce the fixed lingual retainer with attached missing bilateral lateral incisors via the milling technology and using PEEK material.

**Figure 6:**
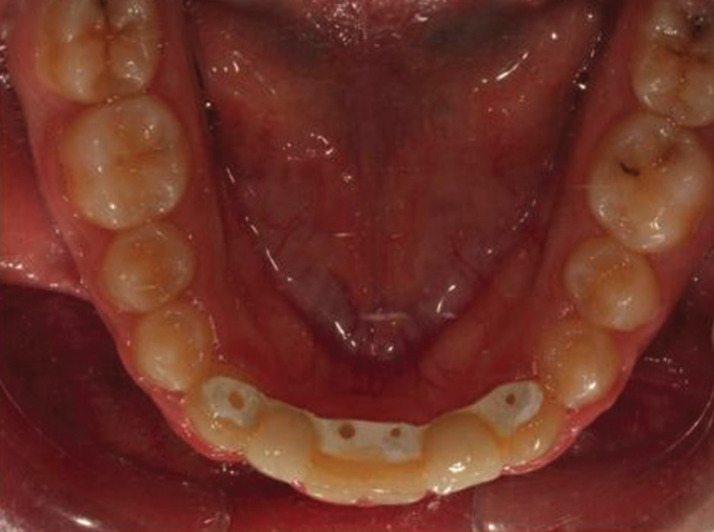
The designed CAD/CAM lingual retainer after orthodontic debonding.

The appliance was delivered clinically with aesthetic satisfaction and occlusion maintenance along with retaining the orthodontic results (Fig. 6).

## Discussion

Within the limitation of this investigation, full digital workflow for lingual fixed orthodontic retainer attached with missing teeth and produced in PEEK material is an acceptable procedure. The digital impression was proven to be precise as conventional along with patient satisfaction (11,12). Nearly all digital orthodontic studies had been related to indirect bonding, lateral cephalometric tracing, model analysis, aligner fabrication, lingual retainer and functional appliances fabrication (4-7). However, the concept of replacing missing teeth with fixed orthodontic retainer was not conducted nor investigated. Thus, long term studies as well as long term complication rates are missing. Successful maintaining of space for possible prosthetic restoration is probably considered important to avoid possible relapse after an orthodontic treatment. Accordingly, it was a main concept in this investigation designing criteria.

The main drawback of this study was the appliance adherence bond breakage. The lack of investigated studies to evaluate the possible bond between PEEK and resin modified glass ionomer cement, leads to further experimental investigations required for evaluating the bonding between PEEK material and resin modified glass ionomer cements is indicated to be able to reduce the risk of debonding.

## Conclusions

1. It’s possible to fabricate lingual retainer via CAD/CAM technology.

2. Digital lingual retainers can be used for replacing missing teeth.

3. PEEK material can be used as a production material for the orthodontic fixed retainer.
